# Preparation and physicochemical characterization of N-succinyl chitosan-coated liposomes for oral delivery of grape seed extract and evaluation of its effect on pulmonary fibrosis induced by bleomycin in rats

**DOI:** 10.22038/IJBMS.2023.70797.15381

**Published:** 2023

**Authors:** Neda Bavarsad, Ali Asghar Hemmati, Fateme Jafarian, Azar Mostoufi, Amir Siahpoosh, Mohammadreza Rashidi Nooshabadi, Esrafil Mansouri

**Affiliations:** 1 Nanotechnology Research Center, Ahvaz Jundishapur University of Medical Sciences, Ahvaz, Iran; 2 Department of Pharmaceutics, Faculty of Pharmacy, Ahvaz Jundishapur University of Medical Sciences, Ahvaz, Iran; 3 Department of Pharmacology, Faculty of Pharmacy, Ahvaz Jundishapur University of Medical Sciences, Ahvaz, Iran; 4 Medicinal Plant Research Center, Ahvaz Jundishapur University of Medical Sciences, Ahvaz, Iran; 5 Marine Pharmaceutical Science Research Center, Ahvaz Jundishapur University of Medical Sciences, Ahvaz, Iran; 6 Department of Medicinal Chemistry, Faculty of Pharmacy, Ahvaz Jundishapur University of Medical Sciences, Ahvaz, Iran; 7 Department of Pharmacognosy, Faculty of Pharmacy, Ahvaz Jundishapur University of Medical Sciences, Ahvaz, Iran; 8 Cellular and Molecular Research Center, Department of Anatomical Sciences, Faculty of Medical Sciences, Ahvaz Jundishapur University of Medical Sciences, Ahvaz, Iran

**Keywords:** Bleomycin, Grape seed extract, Liposomes, N-succinyl chitosan, Pulmonary fibrosis

## Abstract

**Objective(s):**

This study aimed to develop an oral succinyl chitosan-coated liposomal formulation containing grape seed extract and assess its therapeutic efficacy in rats with bleomycin-induced pulmonary fibrosis.

**Materials and Methods:**

N-succinyl chitosan was synthesized, and the liposomal formulations were prepared and characterized regarding phenolic content assay and morphology. Size, zeta potential, in vitro drug release, and stability. Pulmonary fibrosis was induced by intratracheal bleomycin injection, and hydroxyproline measurements, lung weight, animal body weight, as well as histopathological studies were performed

**Results:**

Succinyl chitosan increases the physical stability of the formulation, especially in acidic conditions. Drug release studies revealed that 66.27% of the loaded drug was released from CF2 in an acidic medium in 2 hr, but 92.31% of the drug was released in 8 hr in a pH=7 medium. An *in vivo* study demonstrated that rats exposed to bleomycin significantly lost weight, while those treated with CF2 (400 mg/kg) partially regained weight. Bleomycin treatment increased the mean lung weight and the amount of hydroxyproline in the lungs; these values were significantly decreased in the group treated with 400 mg/kg CF2 (*P<*0.05). Histopathological examination confirmed that treatment with 400 mg/kg CF2 improved lung fibrosis.

**Conclusion:**

In rats, oral administration of N-succinyl chitosan-coated liposomes containing grape seed extract at the 400 mg/kg dose ameliorates bleomycin-induced pulmonary fibrosis.

## Introduction

Pulmonary fibrosis (PF), a fibroproliferative lung disorder (1), is one of the most common and severe forms of idiopathic interstitial pneumonia (2), with a very poor prognosis and median survival time of 2-3 years. Several cytokines and chemokines have been implicated in this process (3).

Bleomycin (BLM) can be used to generate an animal model of PF by inducing DNA breakage and oxidative stress, resulting in an excessive amount of free radicals and abnormal secretion of inflammatory and fibrotic mediators (such as IL-1β, TNF-α, and TGF-β). Subsequently, myofibroblast activation leads to collagen deposition in the lungs (4). The most common but ineffective treatment of PF is corticosteroid administration in combination with immunosuppressive, anti-inflammatory, antifibrosis, antioxidant, and anticoagulant drugs (5, 6). Consequently, studying novel therapeutic agents for the prevention and treatment of PF is in demand. This study aimed to compare the anti-pulmonary fibrosis effects of succinyl chitosan-coated liposomes for oral delivery of grape extract in a bleomycin-induced PF rat model and characterize the liposomes physicochemically.

Grape seed extract (GSE), which contains a high concentration of polyphenolic compounds, may protect against prevalent lifestyle-related diseases such as diabetes and hypertension (7), as well as proliferative, fibrotic, and inflammatory diseases (8). GSE acts as an antioxidant, which may aid in eliminating dangerous free radicals and a variety of pathogenic microorganisms (9).

Liposomes are one of the most extensively studied drug delivery systems due to their ability to encapsulate hydrophobic and hydrophilic active ingredients, biocompatibility, and design flexibility (10). However, numerous barriers exist when liposomes are delivered orally, such as instability in the gastrointestinal tract. By modifying the composition of the lipid bilayers and adding additional ornaments, the stability and permeability of liposomes for oral drug delivery can be enhanced (11, 12). 

Chitosan is a natural and safe polymer with excellent mucoadhesive properties and the capacity of opening tight intercellular junctions of the epithelium (13). N-succinyl-chitosan possesses several advantages, including biocompatibility, low toxicity, and long-term retention in the body, which makes it an attractive drug carrier. Ordinary chitosan is soluble in acidic but not in alkaline water, whereas highly succinylated succinyl-chitosan (degree of succinylation >0.65) has the opposite property (14). The succinyl-chitosan coating may improve the physical stability of the preparations and release of polyphenols in the simulated intestinal environment (15).

GSE was selected as the therapeutic agent for PF in this study, and the liposomal formulation was used to deliver the agent orally. N-succinyl chitosan was used as a coating layer to protect the formulation from the acidic environment of the gastrointestinal (GI) tract and to deliver the active agent to the absorption site.

## Materials and Methods

***Materials***

Soy phosphatidylcholine (Phospholipon® 85 G) was obtained from Lipoid (Germany). Chitosan medium molecular weight was purchased from Aldrich (USA). The dried GSE was purchased from the Jundishapur Incubation Center of Pharmaceutical Technologies (Ahvaz, Iran). Acetone, succinic anhydride, Folin Cioculteu, Na_2_Co_3_, and oleic acid were obtained from Merck (Germany), and Triton X-100 was procured from Acros (USA). HCl, methanol, and NaOH were purchased from the Barad Company in Iran. Male Wistar rats were obtained from the Faculty of Veterinary Medicine, Shahid Chamran University of Ahvaz (Iran).

***N-succinyl-chitosan synthesis***

 Chitosan (0.64 g) was dissolved in an acetic acid solution (5% v/v, 50 ml) and 50 ml methanol was added under stirring. Afterward, 4.22 g of succinic anhydride was dissolved in 30 ml acetone and added dropwise to the chitosan solution. The reaction was stirred overnight at room temperature. Then, NaOH 2M was added dropwise to the viscous gel until a clear solution with a pH of 10 was obtained, then concentrated using a rotary evaporator dialyzed against distilled water for three days and freeze-dried (15). Finally, the N-succinyl-chitosan’s nuclear magnetic resonance (NMR) spectra were recorded.

***Preparation of liposomal formulation***

To prepare formulation 1 (F1), 120 mg soy phospholipid phosphatidylcholine (SPC) was combined with 12 mg oleic acid and 45 mg grape extract, and then 1 ml water was added and completely dispersed, followed by overnight refrigeration to hydrate. The liposomes’ size was reduced using a probe sonicator. To prepare succinyl chitosan-coated liposomes (CF1), an equal volume of liposomes and a 0.5% w/v N-succinyl-chitosan aqueous solution were mixed under gentle stirring. All experiments were conducted under dark conditions (15).

The coated-liposomal formulation 2 (CF2) was prepared as follows: 180 mg (SPC) was combined with 45 mg grape extract and 3 mg succinyl chitosan, then 1 ml water was added, completely dispersed, and refrigerated overnight to hydrate. After 24 hr, the liposomes were reduced in size using a probe sonicator. F2 was prepared in the manner described above but without succinyl chitosan (11). Additionally, blank formulations were prepared and characterized for comparison purposes.

***Characterization of liposomes***

*Phenolic content assay*

Initially, 1.5 ml of liposomes were centrifuged at 15000 rpm for 15 min. The phenolic content of 0.1 ml of supernatant, which did not contain the encapsulated drug, was measured after being diluted with distilled water in a 1:4 ratio. Subsequently,  2.5 ml Folin-Ciocalteu reagent (diluted 1:10) was added to 0.5 ml of the sample and stirred for 2 min before adding 2 ml of Na_2_CO_3_ 7.5% and incubating for 2 hr in the dark at room temperature. Their absorbance was measured at 765 nm. The total phenolic content was determined using a calibration curve developed with tannic acid as a reference (11).

Encapsulation efficiency was calculated using the following formula.

%EE=(Total drug-unloaded drug)/(Total drug)*100

Morphology, zeta potential, and particle size

A Field Emission Scanning Electron Microscope (FESEM) was used to examine the morphology of the prepared formulations. Each sample’s zeta potential was determined in triplicate using dynamic light scattering (Malvern, Nano-ZS). The particle size of the samples was measured in triplicate by laser light scattering (Scatterscope 1, Qudix, South Korea). 

*In vitro drug release*

The dialysis membrane technique was used to conduct the *in vitro* release study. Prior to use, dialysis membranes were hydrated by soaking in distilled water for 20 hr. The formulation (3 ML) was loaded into a dialysis membrane; both ends were sealed and immersed in either simulated gastric fluid (0.1 M HCl, pH 1.2) or simulated intestinal fluid (phosphate buffer solution, pH 7.0) at 37±1 ^°^C while stirring (16). The polyphenols were assessed for 120 min at pH 1.2 and 480 min at pH 7.0. An aliquot of the medium was removed and refreshed at predetermined time intervals to maintain sink conditions. The experiments were performed in triplicate for each preparation (15).

*Stability study*

Formulations were stored at refrigerator temperature (4 ^°^C) for three months. The formulations’ particle size and encapsulation efficiency were determined and compared to the formulations’ initial parameters (16).

**Animal studies**

Animals were kept in separate cages under normal conditions. They had unrestricted access to water and food and 12 hr cycles of light/dark. Thirty male Wistar rats weighing 270-340 g were classified into the following five groups of six:

Group 1 (NS): Received 200 mg/kg of normal saline orally every day for four consecutive weeks.

Group 2 (Ble): One week prior to and three weeks following a single intratracheal injection of bleomycin sulfate at the dose of 7.5 IU/kg, normal saline (200 mg/kg) was administered orally per day.

Group 3 (Ble+100 mg/kg liposomal GSE): One week prior to and three weeks following a single intratracheal injection of bleomycin sulfate at the dose of 7.5 IU/kg, the selected formulation (100 mg/kg) was administered orally per day.

Group 4 (Ble+200 mg/kg liposomal GSE): One week prior to and three weeks following a single intratracheal injection of bleomycin sulfate at the dose of 7.5 IU/kg, the selected formulation (200 mg/kg) was administered orally per day.

Group 5 (Ble+400 mg/kg liposomal GSE): One week prior to and three weeks following a single intratracheal injection of bleomycin sulfate at the dose of 7.5 IU/kg, the selected formulation (400 mg/kg) was administered orally per day.

Animals were anesthetized with 0.3 ml of ketamine hydrochloride (50 mg/ml) intraperitoneally to induce PF. Each rat in the second, third, fourth, and fifth groups received 7.5 IU BLM per kg of body weight through the respiratory tract. Gentian violet (GV) was administered to rats using the same protocol as BLM intratracheal injection to ensure fibrosis induction; the animal was sacrificed, and the lung was examined for color agent receipt (17). 

The body weight of rats was measured on days 1, 8, 15, 22, and 28 of the experiment.

The animals were anesthetized with ketamine at the conclusion of the experiment. The lungs were carefully removed, and the lungs’ weight was determined after a thorough wash with saline. The lung index was calculated by dividing the lung weight by the body weight (18).

For histopathological studies, lung tissues were fixed in 10% formalin and then processed, embedded in paraffin block sections 0.5 mm thick, and stained with Hematoxylin and Eosin (H&E). The sections were examined by light microscopy and assessed for the presence of fibrosis. 

The collagen amount in the samples was determined using hydroxyproline assay based on the Kiazist commercial kit instructions. Briefly, 20 mg of tissue samples were homogenized in 100 µl deionized water and incubated for 4 hr at 4 ^°^C with 12 M HCL in sealed tubes. Each well of a microplate received 20 ml of each sample and standard. The hydrolysates were neutralized with 100 µl of an oxidizing agent (Chloramine T) and incubated for 60 min at 60 ^°^C with chromogen solution. Finally, the samples’ absorbance and standards were determined at 570 nm using a microplate reader (BioTEK, USA). The hydroxyproline concentrations (mg/g tissue) in samples were determined through a standard curve (19).

***Statistical analysis***

The results were expressed as mean±SD. The one-way analysis of the statistical variance test was used to assess the significance of the differences among the various groups. In the case of a significant F-value, the multiple-comparison Tukey’s test was used to compare the means of the different treatment groups. A *P*-value of <0.05 was considered statistically significant.

## Results

***H-NMR and H-H-COSY spectra of N-succinyl chitosan***

**Figure 1:**
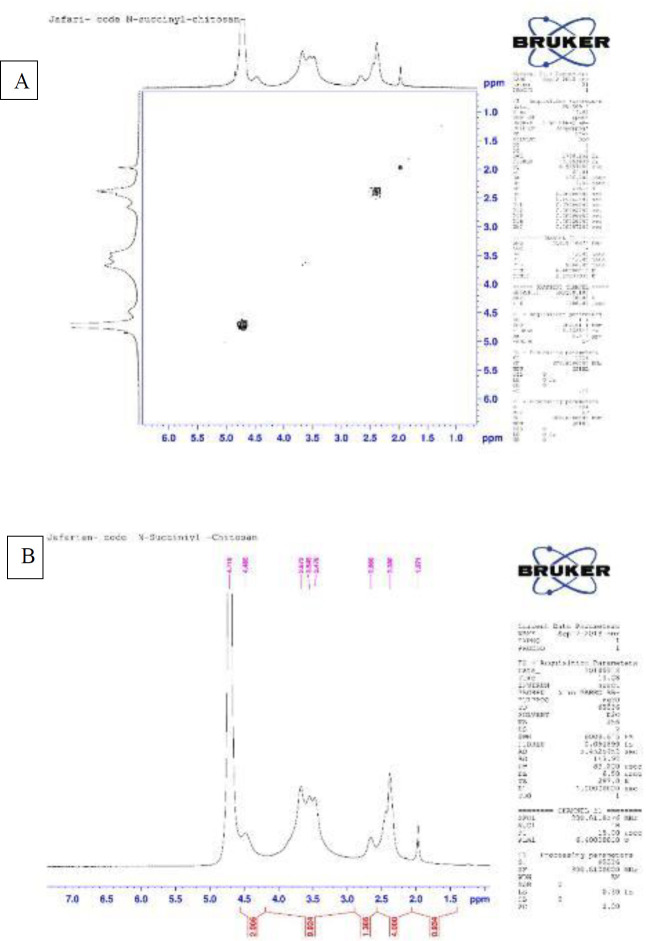
A) H-NMR and B) H-H COSY spectrum of N-succinyl chitosan

Figure 1A illustrates the H NMR spectrum of N-succinyl chitosan. The multiple broad signals at δ=3.47-3.87 (integral 9.924) and the wide and weak signal at δ=2.66 (integral 1.386) corresponded to all the glucosamine ring’s protons. A wide signal at δ=2.38 for the methylene groups in N-succinyl glucosamine (integral 4) confirmed the succinyl glucosamine unit’s existence. The single-point signal at δ=1.97 was potentially related to N-acetyl glucosamine (integral 0.934). The weak signal at δ=4.48 was potentially associated with the anomalous proton. The following ratios were calculated using the four protons of methylene succinyl groups and the integral of the entire ring signals (11.31): N-succinyl glucosamine 53%, N-acetyl glucosamine 16.5%, and glucosamine 30.5%. Figure 1B corroborates the preceding information and is consistent with the relevant sources.

***Characterization of liposomes***

The Tukey HSD test revealed no significant difference in encapsulation efficiency (P>0.05) between the three formulations, F1, F2, and CF1. Although the CF2 formulation had a slightly lower encapsulation efficiency (P<0.05) than the other three formulations, an acceptable level of encapsulation (approximately 90%) was achieved (Table 1).

**Figure 2:**
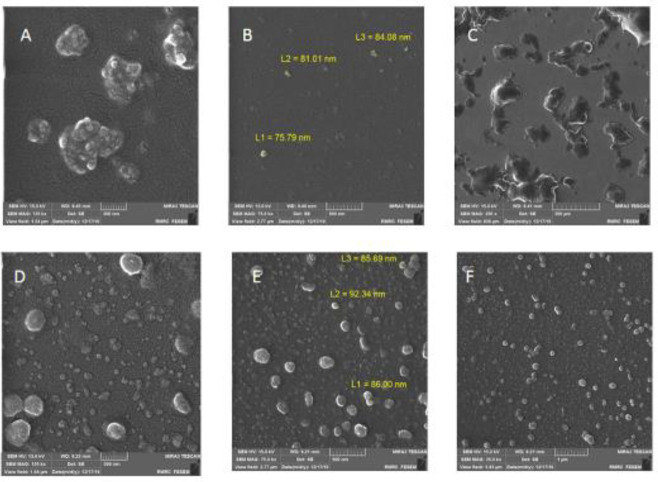
Field emission scanning electron microscopy (FESEM) images of (A, B, C) CF2 and (D, E, F) F2, Scale bar denotes 200 nm (A and D), 500 nm (B and E), 1 μm (F), and 200 μm (C)

FESEM images revealed that the liposomes were spherical and were not destroyed during the coating process (Figure 2).

The zeta potential of liposome formulations measured by DLS is shown in supplemental data. As a result of the presence of oleic acid in the formulation of F1 and CF1, it can be concluded that the sample became heterogeneous. The largest particle sizes corresponded to the coated blank formulations (CB1 and CB2), while the smallest particle sizes corresponded to the F1 and CF1 formulations (Table 1). The Tukey HSD test revealed that formulations F2 and CF2  were not statistically significantly different from one another (*P*>0.05).

*In vitro drug release*

**Figure 3:**
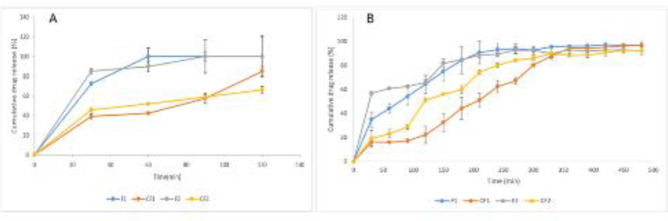
Release profile of phenolic content from different liposomal grape seed extract (GSE) preparations at 37 °C over A) 2 hr at pH 1.2, and B) over 8 hr at pH 7.0 (mean±SD, n=3)

The dialysis method was used to investigate the release behavior of coated and uncoated liposomes at pH 1.2 and 7.0, respectively, and the cumulative release profiles are shown in Figure 3. At pH 1.2, the F1 formulation releases the drug in an upward trend for up to 60 min, at which point 98.90±1.90% of the drug had been released and remained nearly constant. By the end of 120 min, 84.93±4.87% of CF1 had been released. The F1 formulation is significantly different from the CF1 formulation based on the analysis of variance and the Tukey test for drug release at all-time intervals (P<0.001) (Figure 3A).

According to the repeated-measures test results, the drug release from the F2 formulation in an acidic medium reached a maximum in 30 min, when 85.08±2.57% of the drug was released, and there was no significant difference in the amount of drug released after 30 min (*P*>0.05). By the end of 120 min, 66.27±3.23% of the drug had been released from CF2. The analysis of variance and Tukey test revealed statistically significant differences in drug release between the F2 and CF2 formulations at all time intervals (*P*<0.001) (Figure 3A).

At pH 7, the repeated-measures test revealed that drug release from the F1 formulation had an upward trend up to 240 min when almost all drugs (93.04±5.49%) had been released, and there was no significant difference in the amount of drug released after 240 min (*P*>0.05). The CF1 formulation demonstrated an initial delay, with approximately 22% of the drug released in 120 min, followed by a steady release rate from 120 to 360 min, resulting in an almost complete release. According to the analysis of variance and Tukey tests, there was a significant difference in drug release between the F1 and CF1 formulations between 0 and 330 min (*P*<0.05) (Figure 3B). 

F2 released 81.75±3.55% of its content in 150 min, and CF2 released 80.21±2.05% of its content in 240 min. Comparative results of the analysis of variance and Tukey tests indicated a significant difference between drug release from F2 and CF2 formulations for the period between 0 to 240 min (*P*˂0.05) as well as between CF1 and CF2 for the period between 30 to 270 min (*P*˂0.05) (Figure 3B).

*Stability study*

The paired-sample t-test analysis revealed no significant difference in the percentage of encapsulation of liposomal formulations F1, F2, and CF2 after three months of storage (P>0.05). After three months of refrigerator storage, the particle sizes of F1, F2, CF1, CF2, and B1 were significantly different from freshly prepared formulations (P<0.05) (Table 1). 

***Animal studies***

**Figure 4:**
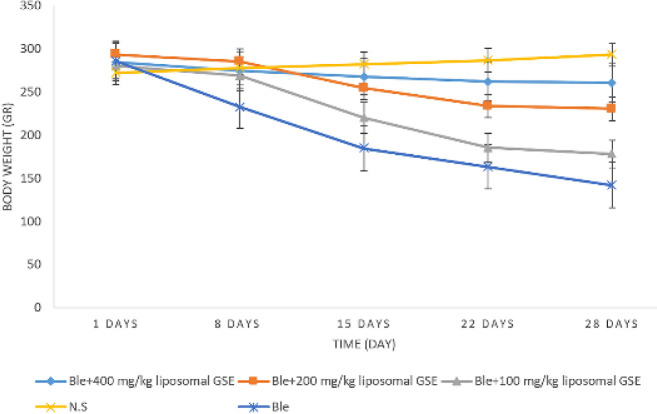
Effect of liposomal grape seed extract (GSE) formulations on rats’ body weight changes (mean+/-SD)

GSE: grape seed extract; Ble: bleomycin

On day 28, the NS group’s mean body weight increased compared to the first day of the experiment. According to the repeated-measures test, weekly weight gain was statistically significant (P<0.001). The mean body weight of groups treated with liposomes was between that of the NS and Ble groups. The Tukey HSD test revealed that body weight was significantly different in the liposome-treated groups than in the Ble group but was not compensated as much as in the NS group (Figure 4).

The lung weights of sacrificed animals were determined at the conclusion of the experiment. The Tukey HSD test revealed that the mean lung weight of the NS group was significantly different from that of the Ble group (*P*<0.001) and from that of the groups receiving 100 and 200 mg/kg liposomal GSE (*P*<0.05). However, there was no statistically significant difference in mean lung weight between the group receiving 400 mg/kg liposomal GSE and the NS group (*P*>0.05) (Table 2). Calculation of the lung index revealed that BLM treatment increases lung weight while decreasing body weight, resulting in a significant increase in lung index compared to the NS group. The lung index of groups treated with liposomal GSE was significantly different from the index of the NS and Ble groups (*P*<0.05) (Table 2).

**Figure 5:**
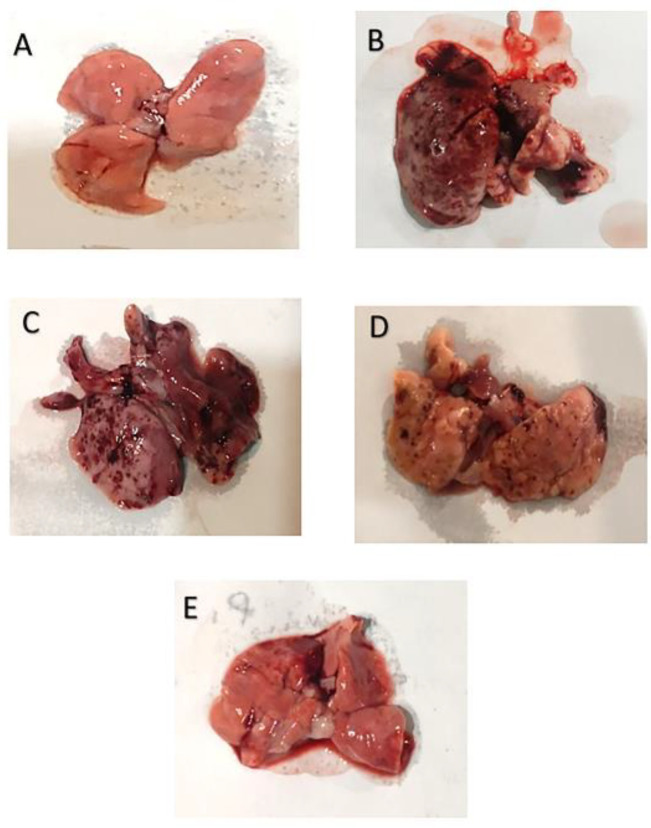
Macroscopic images of rats’ lung

Macroscopic observations of the lungs of animals in the NS group showed that the lungs were normal and pink with suitable elasticity and free of any hemorrhagic damage such as edema and bleeding (Figure 5A). The lungs were swollen and larger than their normal size in the Ble group. Additionally, they exhibited a lack of elasticity. On the lung surface, dark spots caused by hemorrhagic damage were observed (Figure 5B). The liposomal GSE-treated groups demonstrated a low level of fibrotic involvement in comparison to the Ble group. The lungs’ elasticity was significantly improved; an increase in the liposomal GSE intake dose improved the lung’s microscopic evaluation (Figure 5C, D, and E). 

**Figure 6:**
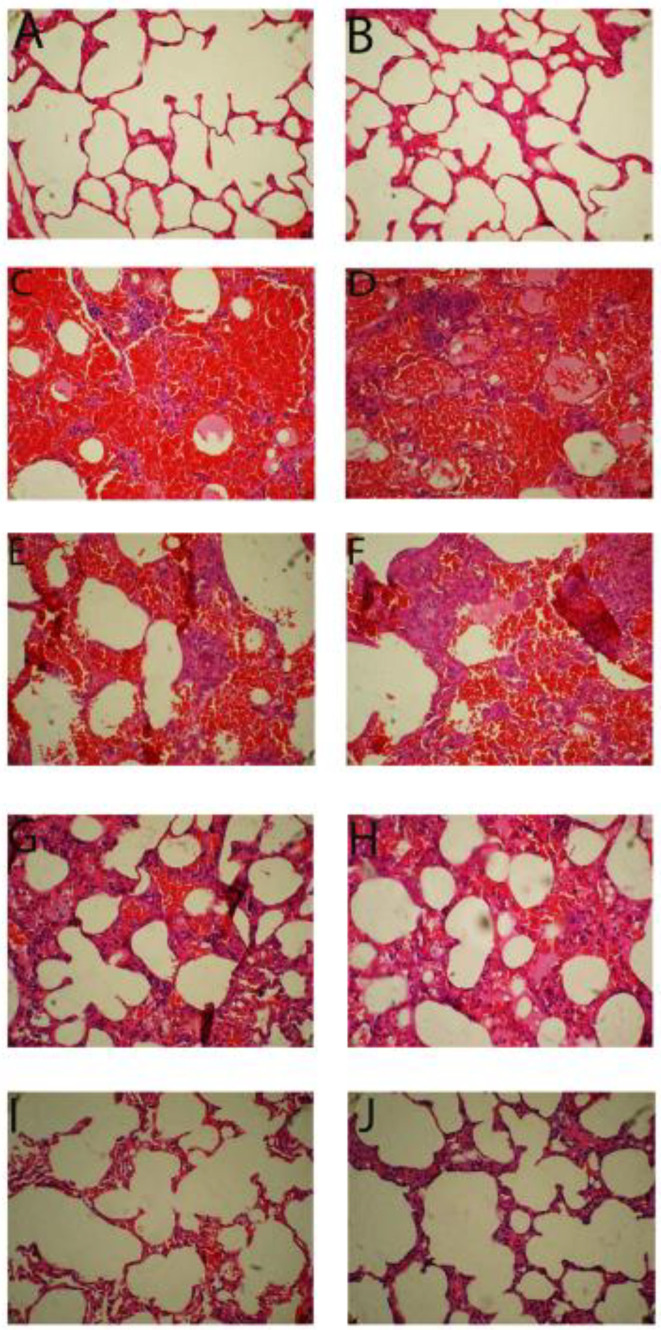
Microscopic images of the rats’ lung (magnification ×300)

Histopathological studies confirmed that the alveoli and the wall between them were normal, and no pathological changes were observed in the NS group (Figures 6A and B). Significant tissue damage was observed in the Ble group. The alveoli reduced in size, the wall between the alveoli thickened, fibrosis was clearly visible, and the wall between the alveoli also bled. Additionally, in most of the organ areas, inflammatory cells were observed in significant numbers (Figures 6C and D). Microscopic images of lung tissue in the Ble+100 mg/kg liposomal GSE group revealed no significant differences in comparison to the Ble group (Figure 6E and F), but a relative improvement in the Ble+200 mg/kg liposomal GSE group was observed, as was an increase in the number of alveoli compared to the Ble group.

Nonetheless, thickened interalveolar walls, bleeding, and a high number of inflammatory cells were still visible (Figure 6G and H). The group receiving 400 mg/kg of GSE seed extract demonstrated a significant improvement over the bleomycin group, with a sharp increase in the number of alveoli, a thinned wall between the alveoli, and the absence of interstitial bleeding and inflammatory cells. The general appearance of this group’s lung tissue was very similar to that of the NS group (Figure 6I and J).

The hydroxyproline assay was used to determine the collagen content of the lungs. A one-way analysis of variance (ANOVA) indicated that the Ble group had a higher hydroxyproline level in lung tissue than the NS group. The Ble group contained the highest concentration of hydroxyproline. The administration of 400 mg/kg liposomal GSE resulted in a significant (*P*<0.05) decrease in the amount of hydroxyproline compared to the Ble group (Table 2).

H-NMR:Proton nuclear magnetic resonance; H-H COSY: Correlated Spectroscopy

**Table 1:** Particle size (nm) and encapsulation efficiency (%) of liposomal grape seed extract (GSE) formulations at time zero (freshly prepared) and 3 months after storage at 4 degree C (mean+/- SD, n=3)

	Freshly prepared formulation		After 3 month storage	
Formulation	Particle size (nm)	% EE	Particle size (nm)	% EE
F1	12.12±1.66	93.42±1.19	435.94±44.72	79.55±6.63
F2	88.67±3.37	93.60±0.96	288.03±50.86	86.90±4.05
B1	228.67±2.06	-	252.03±10.83	-
B2	192.69±3.07	-	203.38±5.41	-
CF1	48.15±1.35	93.07±1.04	378.57±25.21	73.01±4.26
CF2	80.29±4.19	90.40±0.05	257.78±46.22	83.20±3.51
CB1	388.86±2.10	-	396.34±9.15	-
CB2	316.38±6.95	-	344.33±13.35	-

EE: encapsulation efficiency; GSE: grape seed extract; F: formulation; B: blank; CF: coated formulation; CB: coated blank formulation

**Table 2:** Effect of liposomal GSE formulation on rats’ lung weight, lung index, and lung tissue hydroxyproline content (mean±SD)

Groups	Lung weight (gr)	Lung index (mg/gr body weight)	Hydroxyproline (mg/g tissue)
N.S	2.30±0.36	7.98±0.92	2.81±0.28
Ble	5.40±0.96	19.44±4.32	7.65±1.24
Ble+100 mg/kg liposomal GSE	3.60±0.60	13.59±1.54	7.32±0.06
Ble+200 mg/kg liposomal GSE	3.60±0.71	13.16±1.96	6.75±0.05
Ble+400 mg/kg liposomal GSE	3.22±0.46	12.02±0.86	4.89±0.43

GSE: grape seed extract; BLe: bleomycine; NS: normal salin

A) Normal saline group, B) Bleomycin group, C) Ble+100 mg/kg liposomal GSE, D) Ble+200 mg/kg liposomal GSE, and E) Ble+400 mg kg liposomal GSE

GSE: grape seed extract; Ble: bleomycin

A, B) Normal saline group; C, D) Bleomycin group; E, F) Ble+100 mg/kg liposomal GSE; G, H) Ble+200 mg/kg liposomal GSE; and I, J) Ble+400 mg/kg liposomal GSE

GSE: grape seed extract; Ble: bleomycin

## Discussion

PF is a potentially fatal lung disease characterized by the aggregation of extracellular lattice in the interalveolar septum, resulting in an abnormality in the normally functioning lung architecture (5). Despite numerous advances in basic and clinical research, there are currently no effective medical treatments for PF. As a result, developing an effective treatment for PF continues to be a significant challenge (7). We chose to improve the treatment of PF in this study by developing an appropriate formulation. Succinyl chitosan-coated liposomes were tailored to improve the stability and bioavailability of GSE, and the vesicles’ physicochemical properties were determined.

Additionally, empty vesicles were prepared and characterized. We investigated the effect of oral administration of a prepared formulation on bleomycin-induced PF in rats. Notably, while conventional liposomes are rarely used as oral drug delivery systems due to their low resistance to gastric pH and enzymatic degradation, they can be protected by a polymer coating (20).

Chitosan’s various derivatives can interact electro-statistically with negatively charged residues in mucin glycoproteins and provide mucoadhesive properties. In addition, chitosan interacts with a tight junction to facilitate the para-cellular transport of drugs. Moreover, chitosan coating has been considered to increase the membrane integrity and physical stability of liposomes (21).

Most studies that used coated liposomes for oral delivery of a pharmacological agent, concluded that surface modification effectively results in prolonged retention of carriers in the GI tract. Besides Nano-sized small unilamellar vesicles were retained longer than micro-sized multilamellar vesicles in the GI tract (22). Chitosan alone is unable to protect drug-loaded liposomes in the gastric environment because of its high solubility at acidic pH, so mostly its derivatives are used (23).

It is well established that the safety and efficacy of liposomes are contingent upon their stability, which is determined by both formulation and manufacturing techniques. Caddeo *et al*. (2017) synthesized succinyl chitosan-coated liposomes to co-deliver quercetin and resveratrol in the oral cavity. At pH 1.2, the stability of formulations was determined by measuring their size distribution, polydispersity, and zeta potential. The size and polydispersity of succinyl chitosan-liposomes increased slightly, and an inversion of the zeta potential could be due to the presence of H+ protons in the acidic medium. As a result, the succinyl chitosan-liposomes demonstrated excellent resistance to acidic conditions (15). The GSE was encapsulated into vesicles at a greater than 90% rate in our study, with no significant differences between the three formulations, F1, F2, and CF1 (p0.05). The particle size results of liposomes revealed that the largest particle size was found in blank-coated formulations (CB1 and CB2), while the smallest particle size was found in formulations F1 and CF1. 

Liposome morphology analysis using FESEM demonstrated that liposomes are spherical with an average particle size of 88.01 nm; additionally, the coating process did not destroy the liposomes. According to the zeta potential measurements of liposomal formulations, it can be concluded that the presence of oleic acid in F1 and CF1 most likely contributed to the sample becoming non-homogeneous. However, after three months of refrigeration, the drug loading percentage decreased, and the particle size increased, which could be due to the release of some drugs during vesicle fusion. At pH 1.2, F2 (uncoated formulation) rapidly releases its content, reaches a maximum after 30 min, and maintains a nearly constant concentration. However, CF2 (coated formulation) demonstrated lower drug release over a more extended period, indicating that succinyl-chitosan-coated liposomes could sustain drug release in a simulated stomach medium. At pH 7.0, nearly 80% of the polyphenol in CF2 was released in 8 hr at a slower, more steady rate than in the uncoated formulation F2.

There was a gradual gain in body weight in the NS group in the animal study. All Ble-challenged animals lost significant body weight and gained lung tissue weight, followed by a gradual improvement in the liposomal GSE-treated groups. A significant increase in the mean lung/body weight ratio in fibrotic rats was observed. Liposomal GSE treatment significantly decreased this index. Compared to the NS group, the Ble group had severe PF on macroscopic and microscopic examinations. Liposomal GSE treatment significantly reduced bleomycin-induced lung fibrosis when compared to the Ble group, and increasing the liposomal GSE dose resulted in a greater fibrosis-reducing effect, such that the dose of 400 mg/kg resulted in a significant (*P*<0.05) reduction in hydroxyproline amount when compared to the Ble group.

Because the effect of inflammatory and anti-inflammatory cytokines is quite evident in the progressive process of PF, one of the proposed mechanisms of action of GSE in the progressive process of PF could be the antioxidant effects of the flavonoids in this plant, which have the ability to protect against free radical attack in both aqueous and fatty environments, allowing it to be considered an effective antioxidant in the biosphere. Grapes are high in phenolic antioxidants such as phenolic acids, flavonoids, anthocyanins, and tannins (24).

In a study of the protective effect of celery seed on bleomycin-induced PF in rats, Javadi *et al*. (2015) discovered that the bleomycin group had the highest mean hydroxyproline of lung tissue and lung index, while the normal saline group had the lowest. Their study demonstrated that a hydroalcoholic extract of celery reduces the thickness of the alveolar wall and fibrosis progression in mice treated with bleomycin (25).

## Conclusion

This study suggests that the prepared succinyl-chitosan-coated liposomes are an effective oral delivery system for incorporating, protecting, and releasing GSE polyphenols. *In vivo* studies and histopathological findings revealed that liposomal GSE treatment at the 400 mg/kg dose significantly ameliorated PF induced by BLM.

## Authors’ Contributions

N B and AA H designed the experiments; F J performed experiments and collected data; A M designed and supervised the process of synthesis; A S designed and supervised phenolic content assay; E M designed and performed histopathological studies; MR RN collaborated in the animal study; N B, AA H, and F J discussed the results and strategy; N B supervised, directed, and managed the study. All authors approved the final version of the manuscript. 

## Conflicts of Interest

The authors report no conflicts of interest. The authors alone are responsible for the content and writing of this article.
